# Validity arguments for patient-reported outcomes: justifying the intended interpretation and use of data

**DOI:** 10.1186/s41687-021-00332-y

**Published:** 2021-07-30

**Authors:** Melanie Hawkins, Gerald R. Elsworth, Sandra Nolte, Richard H. Osborne

**Affiliations:** 1 Swinburne University of Technology, Centre for Global Health and Equity, School of Health Sciences, PO Box 218, Hawthorn, Melbourne, Victoria 3122 Australia; 2 Charité-Universitätsmedizin Berlin, corporate member of Freie Universität Berlin and Humboldt-Universität zu Berlin, Medical Department, Division of Psychosomatic Medicine, Berlin, Germany

**Keywords:** Validity testing theory, Validation, Health Literacy Questionnaire, Validity, Quantitative research, Qualitative research, Patient-reported outcome measure, PRO, PROM, Health literacy

## Abstract

**Background:**

Contrary to common usage in the health sciences, the term “valid” refers not to the properties of a measurement instrument but to the extent to which data-derived inferences are appropriate, meaningful, and useful for intended decision making. The aim of this study was to determine how validity testing theory (the *Standards for Educational and Psychological Testing*) and methodology (Kane’s argument-based approach to validation) from education and psychology can be applied to validation practices for patient-reported outcomes that are measured by instruments that assess theoretical constructs in health.

**Methods:**

The Health Literacy Questionnaire (HLQ) was used as an example of a theory-based self-report assessment for the purposes of this study. Kane’s five inferences (scoring, generalisation, extrapolation, theory-based interpretation, and implications) for theoretical constructs were applied to the general interpretive argument for the HLQ. Existing validity evidence for the HLQ was identified and collated (as per the *Standards* recommendation) through a literature review and mapped to the five inferences. Evaluation of the evidence was not within the scope of this study.

**Results:**

The general HLQ interpretive argument was built to demonstrate Kane’s five inferences (and associated warrants and assumptions) for theoretical constructs, and which connect raw data to the intended interpretation and use of the data. The literature review identified 11 HLQ articles from which 57 sources of validity evidence were extracted and mapped to the general interpretive argument.

**Conclusions:**

Kane’s five inferences and associated warrants and assumptions were demonstrated in relation to the HLQ. However, the process developed in this study is likely to be suitable for validation planning for other measurement instruments. Systematic and transparent validation planning and the generation (or, as in this study, collation) of relevant validity evidence supports developers and users of PRO instruments to determine the extent to which inferences about data are appropriate, meaningful and useful (i.e., valid) for intended decisions about the health and care of individuals, groups and populations.

## Background

Contrary to common usage in the health sciences, the term “valid” refers not to the properties of a measurement instrument but to the extent to which data-derived inferences are appropriate, meaningful, and useful for intended decision making [37, 99]. Data from patient-reported outcome measures (PROMs) are increasingly being used to inform decisions about, for example, patient care [69, 90], intervention development [3, 20, 57], and pharmaceutical labelling claims [41, 106]. The U.S. Food and Drug Administration (FDA) puts strong emphasis on qualitative evidence in support of the content (i.e., items, domains and scores specific to population, condition and treatment) of an assessment instrument [106] and Edwards et al. explain how a compelling validity argument includes both quantitative and qualitative evidence [37]. However, the predominant focus of validation in health remains on examination of statistical properties of a measurement instrument when psychometrically sound properties alone cannot justify the use of a PROM’s data for any purpose [24, 40, 111]. Validation is not about a static property of a measurement instrument but rather it is a process of obtaining and synthesising validity evidence [7, 111]. The field of health has little to no history of applying modern validity testing theory and methodology to systematically plan for relevant validity evidence to inform the extent to which intended data-based decisions are valid [76, 111]. It would be of use to the field of health to look to the evolution of validity testing theory and methodology in the fields of education and psychology to advance validation practices in measurement of patient-reported outcomes (PRO).

### Validity testing theory

The *Standards for Educational and Psychological Testing* and Michael T Kane’s argument-based approach to validation are the authorities in validity testing theory and methodology, and these draw on a long history of thinking and debating about validity in education and psychology [5–10, 30, 34, 62, 63, 65, 79, 87, 100, 101]. Until about 1950, validity testing theory was based primarily on content and criterion-related validity [100]. Content validity was mainly used in educational testing to confirm the extent to which a test measured specific course subject matter. Test-criterion correlations were used to validate prediction: for example, to decide about someone’s suitability for a job based on their responses to a vocational interest inventory. In the middle of the twentieth Century, a third type of validity, construct validity, came to prominence [10, 33]. In construct validity, test responses are interpreted as a measure of a theoretical construct, which was particularly important for psychology in the early days of working out how to measure personality and intelligence [33, 100]. These three types of validity became the primary model for validity testing [46]. However, problems arose. There were issues with subjective selection of content, confirmation bias, and adequate representativeness of tasks for content validity [45]. Criterion-related validity was problematic for measuring theoretical constructs that had no directly observable and measurable form, and where there was no existing or appropriate criterion measure [32, 45, 100]. It also became clear that these three aspects of validity were interrelated: criterion-related and content validity began to be seen as integral to the evaluation of construct validity. This dilemma led to Samuel J Messick’s Unified Theory of Validation [79].

Messick built on the work of previous theorists [30, 33, 34] to bring the concepts of content and criterion validity together under the heading of construct validity, as the one Unified Theory of Validation [79]. It was Messick’s often-cited 1989 chapter *Validity* in the 3rd edition of *Educational Measurement* [79], and his paper of the same year in *Educational Researcher* [78], that brought the concept to the forefront of validity testing theory. He said that “The essence of unified validity is that the appropriateness, meaningfulness, and usefulness of score-based inferences are inseparable and that the unifying force behind this integration is the trustworthiness of empirically grounded score interpretation, that is, construct validity” (p.5) [78]. The idea of validity being related to the meaning and use of scores, and not to test properties, took some time to take hold but it remained an undercurrent in validity thinking. The argument for this way of conceptualising validity grew over time and was progressively expressed through the different iterations of the *Standards*, from the first technical recommendations in 1954 through to the 2014 *Standards* [5–10]. Worthy of note is the 1985 name change from the *Standards for Educational and Psychological Tests* to the *Standards for Educational and Psychological Testing*, which recognised that the focus of validity is on the process of testing rather than on the test itself [6]. Messick extended the idea of validity to incorporate not just test score meaning but also the value implications of score interpretations, as well as the potential social consequences of testing [80, 82]. Messick’s work remains at the core of validity testing theory today and influenced validity theorists such as Kane whose work strongly influenced the 1999 and 2014 *Standards* [7, 59, 62].

The *Standards* [7] describes the need for evidence based on five sources (Table 1): *test content*; *response processes* of respondents and users; *internal structure*; *relations to other variables*; and the *consequences* of testing as related to a source of invalidity such as construct-irrelevant variance or construct underrepresentation.

**Table 1:** The five sources of validity evidence [7, 49]

**1.**	**Evidence based on test content** The relationship of the item themes, wording and format with the intended construct, and administration including scoring.
**2.**	**Evidence based on response processes** The cognitive processes and interpretations of items by respondents and users, as measured against the intended construct.
**3.**	**Evidence based on internal structure** The extent to which item interrelationships conform to the intended construct.
**4.**	**Evidence based on relations to other variables** The patterns of relationships of test scores to other variables as predicted by the intended construct.
**5.**	**Evidence based on validity and the consequences of testing** Intended and unintended consequences, as can be traced to a source of invalidity such as construct underrepresentation or construct-irrelevant variance.

Depending on the intended measurement purpose, a validity argument will require evidence from one or more of these sources. To apply the validity testing theory of the *Standards* in a practical way, a systematic methodological approach is needed [25–27].

### Validity testing methodology

Kane formalised his practical validity testing methodology in his 1990 essay *An Argument-based Approach to Validation* [25–27, 59, 61, 62, 65]. Following Cronbach [31], the 1985 edition of the *Standards* [6], and Messick [79], construct validity and the interpretive integration of evidence are central to Kane’s approach. There are two steps to Kane’s argument-based approach to validation:

1. The *interpretive argument* (also the interpretation/use argument or IUA): Clearly and coherently state the intended interpretation and use of scores, including the chain of inferences that will lead from observed scores through to decisions based on those scores.
2. The *validity argument*: Evaluate evidence and the construct theory to determine the extent to which these support the interpretive argument.

Kane’s methodology places validation as a process of establishing empirical evidence to build an argument for the validity of the interpretive argument [71]. Interpretive arguments can range from quite simple to more complex depending on what is being measured [25–27, 29, 63]. In his chapter about validation in *Educational Measurement* (p.17–64; 2006) [63], Kane discusses several common interpretive arguments in education including for the measurement of theoretical constructs (p.43), which involves a degree of complexity. Kane explains that an interpretive argument for a theoretical construct relies on five inferences. These inferences come from Stephen E Toulmin’s *practical argument model* [104, 105]: 1) scoring, 2) generalisation, 3) extrapolation, 4) theory-based interpretation, and 5) implications (also called utilisation) [25, 63, 104, 105]. The process of validation involves evaluating the interpretive argument by demonstrating that these inferences are supported by evidence and the theory of the construct being measured. That is, a validity argument explains the extent to which the interpretive argument can be trusted.

Most of the interpretive and validity argument research has been done in language testing and in education. One example is the comprehensive development of a validity argument for the new Test of English as a Foreign Language (TOEFL) [25–27]. This extensive undertaking examined the inferences involved in the TOEFL interpretive argument and provided detailed reasoning and evidence for each inference, and why and how to apply Kane’s methodology [25]. The work of Chapelle et al. required a multi-chapter book to build a validity argument for the TOEFL. Building a full validity argument is usually a cumulative effort and may take many years and multiple studies. The papers by RE Hawkins et al. [52] and Hatala et al. [47] comprehensively outline the development of validity arguments for assessments in medical education and review and collate existing validity evidence. Further, Cook et al. [28] offer advice for applying the *Standards* and Kane to clinical medicine, research and education but they do not develop full interpretive and validity arguments [28]. The 2017 paper of the Montreal Accord on Patient-Reported Outcomes use [99] is an important and detailed review of modern perspectives of measurement validation and PRO scores. The paper references Kane [64] (but not the *Standards*) and discusses the importance of the accumulation of evidence to support the inferences, actions and decisions that arise from the interpretation of PRO scores. But, again, there is no development of interpretive or validity arguments for PRO scores.

### Study rationale

Validity testing theory and methodology are rarely used or even mentioned in validation studies for health assessments [76, 111], yet are recommended generally as best practice for validation planning to avoid piecemeal validity studies that have no focus or synthesis of evidence [37, 111]. Chan’s review (Ch.2, p.21) [111] to determine the extent to which validation standards and guidelines by professional associations and organisations reflect modern views of validity found that the reviewed validation practices tended to consider collection of validity evidence from different sources (or only a single source) as supporting interpretation and use of scores “… without emphasizing the importance of the synthesis of various sources of evidence to support the construct validity of score inferences”. The review by Chan also found that investigation into response processes and consequences (often qualitative research) was recommended in less than half the reviewed standards and guidelines, which is reflected in a review of health literacy assessments where testing of response processes and consequences was rarely conducted [48]. Just as research studies are guided by theory and methodology to understand if, when and how research aims are accomplished (and to guide review of such studies), so do validity testing studies require theoretical and methodological guidance to understand the type of evidence needed and if, when and how the evidence explains the degree to which scores are valid for an intended interpretation and use. It is hoped that this paper will contribute to the rapidly emerging conversation in health measurement about the validity of decisions derived from data (rather than referring to a “valid instrument”) and the types of validity evidence (rather than “types of validity”) that are needed to inform the extent to which data-based decisions are valid.

### Study aim

The aim of this study was to determine how validity testing theory (the *Standards*) and methodology (Kane’s argument-based approach to validation) from education and psychology can be applied to validation practices for patient-reported outcomes that are measured by instruments that assess theoretical constructs in health. The aim will be addressed by developing an interpretive argument and collating relevant validity evidence for a representative theory-based self-report assessment in health. It is expected that the study outcomes will inform validation planning and collation of evidence for the inferences, warrants and assumptions inherent in an interpretive argument. Further analysis, not addressed in this study, is needed to evaluate the merit and strength of the collated evidence and to synthesise it in relation to the inferences in the interpretive argument so as to substantiate the interpretive argument (i.e., build a validity argument).

## Methods

The theory-based self-report assessment used in this study was the Health Literacy Questionnaire (HLQ) [94]. The HLQ is a PRO assessment based on a nine-domain theoretical construct of health literacy. Each scale consists of between 4 and 6 items (44 items in total). The HLQ has two parts. Part 1 (scales 1 to 5) uses four-point response options (score range 1–4): strongly disagree, disagree, agree, and strongly agree. Part 2 (scales 6 to 9) uses five-point response options (score range 1–5): cannot do or always difficult, usually difficult, sometimes difficult, usually easy, and always easy. All items are equally weighted and scores for each scale are summed and divided by the number of items in each scale. Results are the nine scale scores. The HLQ has been used in many settings and for different health conditions, including public and private hospitals [55, 56]; health professional students in universities [88, 103]; Indigenous people with chronic disease in remote Australia [97]; community-based clients with diabetes receiving home nursing services [42]; migrant populations [35, 98]; rural fishing villages in Egypt [11]; and cardiac rehabilitation [2].

In our expository study, Kane’s five inferences for theoretical constructs were applied to the existing general interpretive argument for the HLQ [94]. The HLQ development paper provides a broad statement about the intended interpretation and use of HLQ scores: “We sought to develop an instrument that was capable of detecting a wide range of health literacy needs of people in the community, and that could be used for a variety of purposes from describing the health literacy of the population in health surveys through to measuring outcomes of public health and clinical interventions designed to improve health literacy” (p.2) [94]. Based on this statement and the use of the HLQ in studies using the Ophelia (Optimising Health Literacy and Access) process [16, 17], HLQ data are intended to be interpreted as profiles (based on the nine scale scores) of health literacy strengths and needs and used for a range of purposes, such as:

1. describing the health literacy of populations
2. informing foci for health literacy interventions
3. measuring outcomes of public health and clinical interventions designed to improve health literacy.

The indicators of the nine-domain theoretical construct of the HLQ (Table 2) are the 44 items, which are calculated as nine scale scores [94]. Inferences are based on assumptions that the warrants (i.e., the grounds or rules) on which an inference is made are backed by evidence [7, 12, 25–27, 63, 83, 84, 105]. The extent to which an interpretive argument can be justified (i.e., the degree to which it is valid) depends on the generation of corroborating evidence and the detection of threats (i.e., rebuttal arguments). If any one of the five inferences cannot be supported by evidence then justification of the inferences in relation to the construct theory is uncertain and the validity of the interpretive argument is questionable, perhaps invalid [33, 63, 65]. The sources of evidence, warrants and assumptions associated with Kane’s five inferences are described in relation to the HLQ in Table 2.

**Table 2:** The five inferences for measurement of theoretical constructs as related to the Health Literacy Questionnaire (HLQ)

**1. Scoring inference**	The scoring inference assumes that users of the HLQ will abide by the warrant of the HLQ scoring instructions. The evidence for this inference is derived from development information about the HLQ items, scales and response options and scoring procedures, which includes that scoring is free from bias [7, 63, 94]. Statements about how a study scores a PROM need to be clear because this provides evidence for the assumption that the scoring has taken place as intended. The validity of the scoring inference is the basis for the validity of all other inferences.
**2. Generalisation inference**	The warrant for the generalisation inference follows the same principle of any generalisation study: that scores are estimates (representative) of the scores that other similar respondents (from a universe of possible respondents) would get on the same or similar measure (e.g., a translated HLQ). The assumption is that context is not relevant to scale score interpretations (i.e., that time, place, language/culture or other contextual factors do not present validity threats). The *Standards* states that evidence for generalisation (relations to other variables) stems from meta-analyses and statistical summaries of past studies (e.g., cumulative databases) (p.18) [7]. While reliability evidence is relevant to every inference, it is predominantly applicable to the generalisation inference [47], and is reported as such in this study.
**3. Extrapolation inference**	The extrapolation inference is the first step in the process of linking the observed HLQ scores to the nine-domain health literacy theory. It is this inference that underpins the majority of psychometric “construct validity” testing in health measurement. The warrant for this inference is that the HLQ scale scores are accurate representations of the corresponding HLQ health literacy domains (i.e., the target scores). This warrant assumes that the nine scale scores account for a range of attributes, resources and competencies that people need for accessing, understanding, and using health information and services to manage their health [94]. The evidence for this inference [7] includes: • information derived from the processes used to develop the HLQ items, scales and response options, and how respondents interpret and understand these; • the internal structure of the HLQ domains using methods that, for example, test if response patterns conform to the nine scale scores; • and the relationships between the HLQ scales, which while related, are distinctly independent from each other.
**4. Theory-based interpretation inference**	The warrant for the theory-based interpretation inference is the nine-domain health literacy theory, which is operationalised in the high and low score descriptions [94]. This warrant assumes that the HLQ health literacy theory (i.e., domain descriptions) explains the scales and items, and that the item and scale scores provide appropriate estimates of the theory [63]. The evidence for this inference is derived from evaluation of the content of the HLQ items and how respondents engage with the items [7].
**5. Implications (or utilisation) inference**	The overarching warrant for the implications (or utilisation) inference is the rules for decisions based on HLQ data. Bachman (pp.18–20) [12] describes four specific warrants for a data utilisation argument: • Relevance: that the score-based interpretation is relevant to the decision to be made. • Utility: that the score-based interpretation is useful for making the intended decision. • Intended consequences: that the consequences of using the assessment and making intended decisions will be beneficial to individuals, the program, company, institution, or system, or to society at large. • Sufficiency: that the assessment/s provide sufficient information for making the decision. The main assumption underlying these four warrants is that the HLQ health literacy construct (operationalised through the nine scale scores) embodies several factors (the nine theoretical domains) that influence health outcomes (and potentially health equity). The evidence for this assumption is based primarily on the validity-related consequences of data-based decisions [7, 12, 54, 58, 63, 72].

After establishing a general interpretive argument, a literature review was conducted to find existing validity evidence for the HLQ. HLQ validity testing studies were collated by the first author (MH) through a search of the peer-reviewed literature in EBSCOhost databases: Academic Search Complete; CINAHL Complete; ERIC; Global Health; MEDLINE Complete; PsycINFO. No limiters were applied. Articles with statements in the title or abstract about undertaking validity testing of the HLQ were included for data extraction. Reference lists of pertinent systematic reviews were scanned for relevant articles, as were article reference lists and the authors’ personal reference lists. This study categorised HLQ data only as sources of validity evidence, as defined by the *Standards*. Reliability evidence and general study characteristics were also extracted from the articles. A descriptive and frequency analysis approach was used to identify, categorise and count the sources of evidence [48, 50]. The reliability of data extraction and categorisation was ascertained through extensive consultations between MH and GE and with frequent reference to the descriptions of the five sources of evidence in the 2014 *Standards*. The collated sources of validity evidence were then aligned with the five inferences (and underlying warrants and assumptions) of the HLQ interpretive argument, as specified by the *Standards*.

## Results

**Fig. 1:**
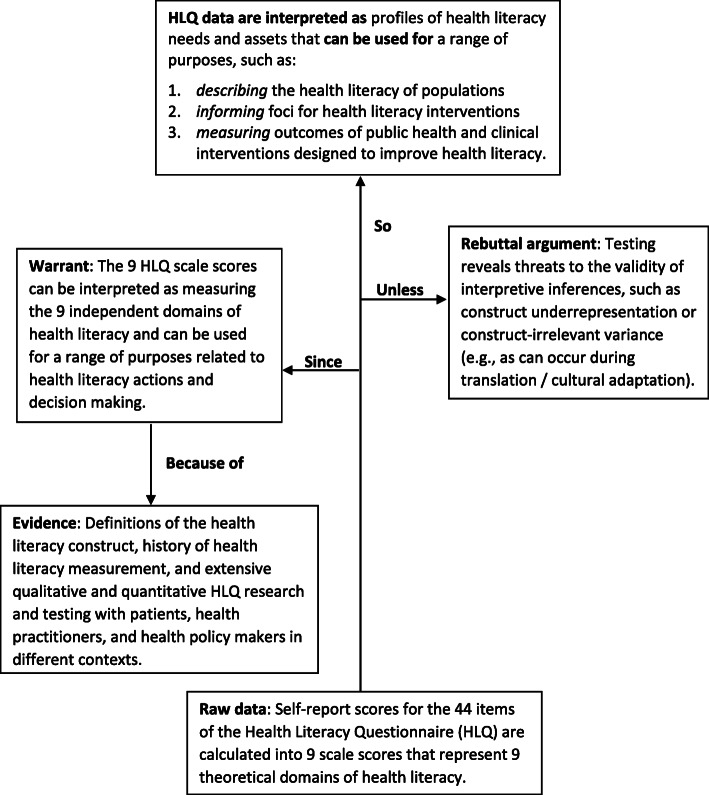
A general interpretive argument for the Health Literacy Questionnaire

Figure 1 displays the general HLQ interpretive argument [25–27, 104, 105]. The central vertical arrow denotes Kane’s five inferences for theoretical constructs that connect the data (HLQ raw scores) to the intended interpretation and use of the data [94]. The five inferences rely on one overarching warrant that the nine independent HLQ scale scores can be interpreted as measuring the nine theoretical domains of health literacy and are appropriate for the intended use, as backed by evidence and with acknowledgement of potential threats to the inferential pathway.

**Fig. 2:**
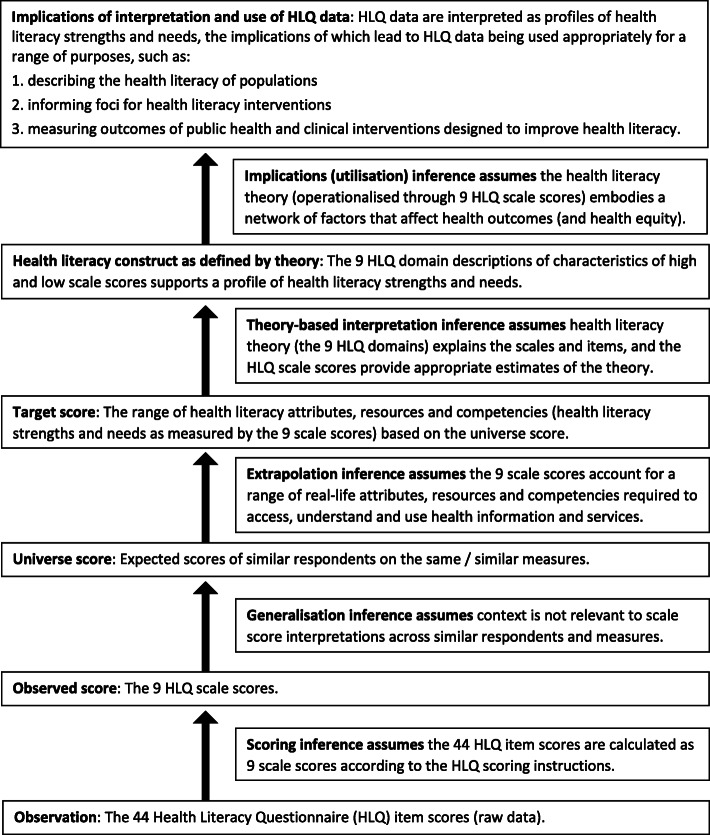
Theory-based inferences and the warrants and assumptions of the general HLQ interpretive argument

Figure 2 shows the connections between the warrants and assumptions and each of the five theory-based inferences of the HLQ [63], which are denoted by the central vertical arrow in Fig. 1 [25]. Figure 2 is read from the bottom to the top – from the observation of raw data up through the assumptions, warrants and inferences that lead to the implications of the interpretation and use of HLQ data. The HLQ raw data are calculated into observed scores on the assumed warrant of the *scoring inference* that scores are calculated as per the HLQ scoring instructions. Next, the *generalisation inference* assumes the warrant that scale scores are representative of a universe of scores from other similar respondents and measures. The *extrapolation inference* links data to theory and assumes the warrant that the nine scale scores are representative of the nine health literacy domains (e.g., scale scores account for real-life attributes, resources and competencies). The *theory-based interpretation inference* assumes the warrant that a relationship exists between the nine-domain theory and the items and scales such that the HLQ scores support profiles of health literacy strengths and needs. Finally, the *implications (or utilisation) inference* assumes the warrant that the nine theoretical health literacy domains embody factors that affect health outcomes. Evidence to support each of these inferences implies that HLQ data (in the form of the nine scale scores) can be used appropriately and meaningfully for the intended purposes listed in the top row of Fig. 2.^1^

The review resulted in 11 articles in which validity testing of the HLQ had been conducted [19, 36, 39, 43, 51, 53, 68, 74, 86, 92, 94]. Table 3 displays the countries in which these studies were conducted, the years of publication, and the evidence for reliability.

**Table 3:** Country and year of publication, and reliability evidence

Country of study	N
Australia	5
China	1
Denmark	2
France	1
Germany	1
Slovakia	1
*Total*	*11*
**Year of publication**	**N**
2013	1
2014	1
2016	2
2017	4
2018	2
2019	1
*Total*	*11*
**Reliability**	**N**
Cronbach’s alpha	8
Composite reliability	6
Inter-rater reliability	1
Item response theory	1
Person Separation Index	1
*Total*	*17*

Table 4 displays the number of times the five sources of validity evidence were reported across studies. In total, validity evidence was reported 57 times, with evidence based on *test content* reported the most frequently (*n* = 23; 40%). There were no studies that reported evidence about the *consequences* of testing.

**Table 4:** Sources of validity evidence reported for all studies

Evidence based on	N
1. Test content	23
2. Response processes	4
3. Internal structure	15
4. Relations to other variables	15
5. Validity and the consequences of testing	0
*Total*	*57*

A theoretical validity testing framework was referenced by four of the 11 HLQ papers [39, 43, 51, 94], with statements to support the citations: one paper [39] directly referenced Messick [4]; another paper [51] directly referenced Messick [81], Kane [62] and the *Standards* [5]; and two papers [43, 94] indirectly referenced the *Standards* through Hawkins et al. (2018) [43, 49] and Buchbinder et al 2011 [23, 94].

Table 5 displays the interpretive argument of the HLQ (Fig. 1), including the five inferences and the warrants and assumptions underlying each inference (Fig. 2), and the corresponding sources of validity evidence for the HLQ.

**Table 5:** The inferences, warrants and assumptions of the general HLQ interpretive argument, and existing sources of validity evidence

Inferences	Warrants	Assumptions	Sources of validity evidence
1. Scoring	HLQ scoring instructions.	The 44 HLQ item responses are calculated as nine scale scores according to the HLQ scoring instructions.	*Test content*: all 11 studies
2. Generalisation	The nine HLQ scale scores are estimates of scores of other similar respondents across HLQ versions / similar measures.	Context is not relevant to scale score interpretations across similar respondents and measures.	*Relations to other variables*: no meta-analyses or accumulated data studies were found. *Reliability*: [19, 36, 39, 43, 53, 68, 74, 92, 94]
3. Extrapolation	HLQ scales represent the elements of the nine HLQ domains.	The nine scale scores account for a range of attributes, resources and competencies required to access, understand and use health information and services.	*Test content*: [19, 36, 51, 53, 74, 92, 94] *Response processes*: [51, 74, 94] *Internal structure*: [36, 39, 43, 53, 68, 74, 86, 92, 94] *Relations to other variables*: [19, 36, 39, 53,74, 92, 94]
4. Theory-based interpretation	The health literacy theory: descriptions of high/low scores of the nine HLQ conceptual domains.	Health literacy theory (the nine HLQ domain descriptions) explains the scales and items, and the HLQ scale scores provide appropriate estimates of the theory.	*Test content*: [92, 94] *Response processes*: [51, 74, 94]
5. Implications (utilisation)	Decision rules (p.28) [33]: The attributes, resources and competencies represented by the nine HLQ domains are relevant to improving health outcomes. Data from the HLQ are useful for informing decisions about health literacy practice and policy. The consequences of using scale scores to make practice and policy decisions will result in the intended benefits. HLQ data are sufficient for making these decisions (or there is need for additional information) (p.18) [87].	The health literacy construct (operationalised through HLQ scale scores) embodies a network of factors that affect health outcomes (and health equity).	*Consequences*: No studies were located.

### 1. Scoring inference – HLQ sources of evidence

The *Standards* places information about scoring in *test content* [7]. All 11 studies stated the way that the HLQ was scored. The HLQ scoring instructions were written in the development stage and introduced in the original HLQ development paper [94]. The study by Kolarčik et al. [68] conducted testing on two sets of wording for the Part 2 response options of the HLQ: the original and revised versions and produced evidence in support of the revised response options.

### 2. Generalisation inference – HLQ sources of evidence

In the HLQ studies, reliability was calculated mainly through Cronbach’s alpha with or without composite reliability (*n* = 9 studies) [19, 36, 39, 43, 53, 68, 74, 92, 94]. Kolarčik et al. [68] also calculated reliability using IRT. Morris et al. [86] used the Person Separation Index (PSI), and Hawkins et al. [51] used a qualitative inter-rater reliability method to examine concordance (and discordance) between patient- and clinician-reported HLQ scores and interview narratives. No HLQ generalisation studies based on meta-analyses or accumulated data were located.

### 3. Extrapolation inference – HLQ sources of evidence

Evidence for extrapolation from observed data to a theoretical construct can be extensive and is based on four of the five sources of evidence in the *Standards*: *test content*, *response processes*, *internal structure*, and *relations to other variables*.

Evidence based on *test content* included information about item content and difficulty (as described by the HLQ item intent descriptions) [19, 36, 51, 53, 74, 92, 94], response options and scoring (although this is discussed under the scoring inference), and administration formats. Methods included expert review [92, 94]; involvement of the target audience (HLQ respondents and users) in the development of scales and items [94]; development of construct high/low and item intent descriptions [94]; comparison of respondent narratives with item intent descriptions [51]; examination of administration methods [94]; and item difficulty studies [19, 36, 53, 74, 94].

Evidence based on *response processes* provided analyses about what the items and scales mean to respondents and users of the HLQ (i.e., how they think about, interpret and respond to the items, given their life experiences) [112], and whether or not these meanings conform to the intended theoretical construct of health literacy [95]. Three studies investigated the ways respondents cognitively engaged with the HLQ items. All studies used the method of cognitive interviewing [51, 74, 94]. One of the studies was counted as producing two instances of evidence based on *response processes* because the interviews were conducted with both users of HLQ data (clinicians) and respondents (patients) [51]. The only study of a translated HLQ to report investigation of *response processes* was the Maindal et al. study, which conducted cognitive interviews.

Evidence based on *internal structure* described the interrelationships of items within scales, and the extent to which the items conform to the construct of that scale [77]. Some studies used more than one method to conduct internal structure analyses resulting in 15 instances of evidence based on *internal structure*. Confirmatory factor analysis (CFA) [36, 39, 43, 53, 68, 74, 92, 94] and differential item functioning (DIF) [68, 86] studies were primarily used (*n* = 9 studies). The Morris et al. study also used principal component analysis (PCA) and Rasch analysis [86] and Osborne et al. also used item-remainder correlations and intra-factor correlations [94].

Evidence based on *relations to other variables* is usually derived from studies that analyse the relationships between an instrument and other criteria [77]. However, for a multidimensional instrument such as the HLQ (nine scales), there must be evidence that each of the scales is unidimensional and distinct from the other scales (pp.38–39) [77]. The nine HLQ scales, although related, are intended to measure different aspects of the health literacy construct. The *Standards* places evidence for discriminant validity within *relations to other variables* (p.16) [7], so this is how the HLQ studies that calculated discriminant validity between the nine scales were categorised (*n* = 7 studies) [19, 36, 39, 53, 74, 92, 94]. Criterion-referenced validity was investigated by Goodwin et al. [43] using linear regression models and the SF-36 [22, 109] to test the strength of associations between each of the nine HLQ scales and physical and mental health. Huang et al. [53] did correlation studies with the SF-12 [110] using the Spearman correlation coefficient. There were five studies that examined group differences (i.e., relationships between the HLQ scales and background characteristics such as demographic information) [19, 36, 39, 68, 74].

### 4. Theory-based interpretation inference – HLQ sources of evidence

Evidence for the theory-based inference can also rely on evidence based on *test content*, *response processes*, *internal structure*, and *relations to other variables*. However, instead of the focus being on whether or not the scale scores account for the scope of the health literacy construct (extrapolation inference), the focus for the theory-based interpretation inference is on evidence to support scale scores as estimates of the theory. That is, the focus is on evidence for the extent to which a scale score estimates a person’s or population’s level of attributes, resources or competencies associated with that HLQ domain. Evidence, therefore, relies heavily on *test content* and *response processes* to understand the cognitive connection between the HLQ domains and the items and scales. Often these are qualitative studies undertaken during development (e.g., expert review, and information feedback or cognitive interviews with patients, community members or users) [51, 74, 92, 94]. The HLQ authors wrote item intent descriptions (i.e., descriptions of the meaning and difficulty), which are useful for determining if respondents are engaging with the items as intended [94]. The item intents and domain descriptions are particularly useful for translation or adaptation studies where equivalence of meaning and measurement is essential for the integrity of score interpretation and use within and across cultures [68, 74, 92].

### 5. Implications (or utilisation) inference – HLQ sources of evidence

Evidence to support this assumption is based on the *consequences* of using the data to inform decisions and actions [7, 12, 54, 63, 72]. The Ophelia (Optimising Health Literacy and Access) process uses HLQ data in needs assessments to support communities (e.g., healthcare organisations, community healthcare, local and federal governments) to identify health literacy needs and assets. Interpretations of these data have influenced decisions about interventions and health system improvements [1, 13, 14, 16–18, 42, 57, 66, 67] but evidence of the extent and, particularly, the effectiveness of this use of HLQ data in these and other contexts is needed. Comprehensive pre-post evidence of the outcomes of health literacy interventions informed by HLQ data are still being generated.

### Gaps in validity evidence

Validation is an ongoing process of checking that the interpretation and use of a PRO assessment’s data are valid for each new context, and so generation of validity evidence for the use of HLQ data in different contexts is ongoing. There is incomplete evidence for the generalisation (except for reliability) and implications inferences. Also, the HLQ has been translated to more than 30 languages since 2013 but evidence based on *response processes* (how people engage with, think about, interpret, and respond to the items) was found in only one HLQ translation study [74]. Although it is known that other response process testing has taken place, this evidence has not been published. This is the most obvious gap in evidence required to support the adequacy and appropriateness of HLQ score interpretation and use in other language and cultural contexts.

## Discussion

This study integrates validity testing theory and methodology from education and psychology with measurement research in health. Kane’s five inferences and associated warrants and assumptions were demonstrated in relation to the HLQ, a PROM based on a theoretical construct. Existing HLQ validity evidence was collated within the *Standards*’ five sources of validity evidence and mapped to the five inferences in the HLQ interpretive argument. Further research is required to synthesise and evaluate the evidence to determine a validity argument for the HLQ general interpretive argument, and for each stated purpose of HLQ data. Table 5 provides the basis for future validity testing research in health measurement for the evaluation of evidence for validity arguments in relation to the five inferences for PROMs that assess theoretical constructs in health.

Collation and categorisation of existing sources of validity evidence for the HLQ shows that some sources of evidence have been published for HLQ scoring, extrapolation and theory-based interpretation inferences but minimal evidence is available for the generalisation and implications inferences. The HLQ is a relatively new measurement instrument that has been quickly taken up for use in a wide range of studies in more than 60 countries. The development and publication of validity evidence has not kept pace with the application of the HLQ in practice. However, since the literature review for this study was conducted, more articles presenting evidence for the HLQ have been published [1, 2, 11, 13, 21, 66, 73, 89, 96, 107, 108].

For translations of the HLQ, gaps mainly exist for evidence based on *response processes*. Many HLQ translation teams did conduct cognitive interviews but the processes and outcomes of this testing have not been published. Evidence based on *internal structure* has been generated for translated HLQs through CFA to confirm that the relationships between HLQ items and scales in different contexts behave in comparable ways to the English-language HLQ items and scales. However, full analyses of measurement invariance comparing data from translated and original versions of the HLQ using, for example, multiple-group confirmatory factor analysis are lacking. This type of analysis is essential if cross-cultural data comparisons are to take place [14]. Evidence based on *relations to other variables* is common for the HLQ because of the decision in this study to categorise calculations of discrimination between each of the nine scales under discriminant validity: that is, although the nine scales represent the overall construct of health literacy, analyses should show that each scale is distinct from the other scales. However, it should be noted that there are limited studies of convergent and discriminant validity with other constructs in the nomological network of health literacy.

### Validation planning

The findings of this study are important because they demonstrate that validity testing theory and methodology from education and psychology can be applied to the development, testing, and retrospective evaluation of a theory-based health literacy measure, and can potentially be applied to other self-report PROMs [15, 85]. This study retrospectively collated existing sources of validity evidence to inform the direction of future HLQ validity studies and research. However, the findings from this study can also be used to prospectively inform the types of evidence needed to determine the extent to which data from new PROMs are valid [15]. Invalid data can lead to ill-informed decisions about people’s health [70, 111]. If any one of the inferences in an interpretive argument is not supported by evidence, then the data are invalid. For example, the HLQ scoring inference assumes that users will score the HLQ as intended. If not, then all subsequent inferences are implausible, and the data are invalid and cannot be used for the intended purpose. There is currently no validity evidence to support alternative scoring strategies for the HLQ, such as developing a single total score. Messick emphasised that the appropriateness, the meaningfulness and the usefulness of score-based inferences must be evaluated, and this is essential for determining how decisions based on PROM data will benefit or perhaps disadvantage people [58, 75, 82, 91].

### Quantitative and qualitative research address different aspects of validity testing

Quantitative analyses can confirm the psychometric properties of an instrument, but these data do not explain how people interpret and cognitively respond to items or the consequences of data-based decisions [54, 58, 70]. For example, during the translation process of a PROM from one language to another, there is a risk of unintentionally jeopardising measurement equivalence through introducing construct-irrelevant variance or of not noticing that there is construct underrepresentation in the new culture [38, 44, 49]. Qualitative research methods are used to investigate the transference of *test content* to another language and to understand the *response processes* of the target population when they engage with the translated instrument’s items [24, 60, 93, 95, 102]. The use of validity testing theory and methodology in PROM validation practice enables systematic approaches to generating the sources of evidence that are most important for the PROM’s intended score interpretation and use [25, 111].

### Limitations and strengths of this study

A limitation of this study is that it does not evaluate existing HLQ validity evidence to develop a validity argument for the HLQ or to determine gaps in evidence to support HLQ data for decision making in different contexts and for different purposes. Future research for the HLQ will involve more detailed analyses of the outcomes of the studies reviewed. The predominant strength of this study is that it demonstrates that well-developed theory and methodology from education and psychology can be applied to validation practice for PROMs. This study was retrospective in nature, but the process can also be used prospectively for validation planning and testing of newly developed PROMs.

## Conclusions

PROM validation practice needs to evolve from referring to validity as a property of a measurement instrument to understanding that validity refers to the data-derived inferences that influence decision making. This study demonstrated a process for systematic and transparent validation planning (i.e., build an interpretive argument outlining inferences, warrants and assumptions) and collation of relevant existing validity evidence, using the HLQ as an example PROM. Such a process enables PROM developers and users to then synthesise evidence and evaluate the extent to which the evidence supports the appropriateness, meaningfulness and usefulness (i.e., the validity) of the data for making decisions about the health and care of individuals, groups and populations in different contexts.

## Data Availability

All data generated or analysed during this study are included in the article.

## Footnotes

1 Note that different types of evidence may be required to support the use of HLQ scale scores for different potential purposes (see the top row of Figure 2). This study provides an *example* of how to use Kane’s five inferences to develop an overall interpretive argument for a theory-based self-report questionnaire. It also shows, as a general example, how to align the five sources of validity evidence from the *Standards* with each inference. Future studies are required to define specific interpretive arguments for specific uses of the data and outline or provide the evidence required to determine the extent to which the interpretive argument is valid.

## References

[1] Aaby A, Beauchamp A, O’Hara J, Maindal HT (2020). Large diversity in Danish health literacy profiles: Perspectives for care of long-term illness and multimorbidity. European journal of public health.

[2] Aaby A, Friis K, Christensen B, Maindal HT (2020). Health literacy among people in cardiac rehabilitation: Associations with participation and health-related quality of life in the heart skills study in Denmark. International journal of environmental research and public health.

[3] Aaby A, Simonsen CB, Ryom K, Maindal HT (2020). Improving organizational health literacy responsiveness in cardiac rehabilitation using a co-design methodology: Results from the heart skills study. Int J Environ Res Public Health.

[4] Alkin MC (1992). Encyclopedia of educational research.

[5] American Educational Research Association, American Psychological Association, Joint Committee on Standards for Educational and Psychological Testing (U.S.), National Council on Measurement in Education (1999). Standards for educational and psychological testing.

[6] American Educational Research Association, American Psychological Association, National Council on Measurement in Education (1985). Standards for educational and psychological testing. American Educational Research Association.

[7] American Educational Research Association, American Psychological Association, National Council on Measurement in Education (2014). Standards for educational and psychological testing.

[8] American Psychological Association, American Educational Research Association, National Council on Measurement in Education (1954). Technical recommendations for psychological tests and diagnostic techniques, vol 51. vol 2. American Psychological Association.

[9] American Psychological Association, American Educational Research Association, National Council on Measurement in Education (1974). Standards for educational & psychological tests. American Psychological Association.

[10] American Psychological Association, American Educational Research Association, National Council on Measurement in Education, American Educational Research Association Committee on Test Standards (1966). Standards for educational and psychological tests and manuals. American Psychological Association.

[11] Anwar WA, Mostafa NS, Hakim SA, Sos DG, Abozaid DA, Osborne RH (2020). Health literacy strengths and limitations among rural fishing communities in Egypt using the health literacy questionnaire (HLQ). PLoS One.

[12] Bachman LF (2005) Building and supporting a case for test use. Language Assess Quarter 2 (1):1–34, DOI: 10.1207/s15434311laq0201_1

[13] Bakker, M., Putrik, P., Rademakers, J., Van de Laar, M., Vonkeman, H., Kok, M., … Osborne, R. (2020). OP0257-PARE using patient health literacy profiles to identify solutions to challenges faced in rheumatology care. *Ann Rheum Dis*, *79*(Suppl 1) 162.2, 16162. 10.1136/annrheumdis-2020-eular.877.

[14] Bakker MM, Putrik P, Aaby ASE, Debussche X, Morrissey J, Borge CR, Kolarcik P, Batterham R, Osborne RH, Maindal HT (2019). Acting together–WHO National Health Literacy Demonstration Projects (NHLDPs) address health literacy needs in the European region. Public Health Panorama.

[15] Barnett LM, Mazzoli E, Hawkins M, Lander N, Lubans DR, Caldwell S, Comis P, Keegan RJ, Cairney J, Dudley D (2020). Development of a self-report scale to assess children’s perceived physical literacy. Physical Education and Sport Pedagogy.

[16] Batterham RW, Buchbinder R, Beauchamp A, Dodson S, Elsworth GR, Osborne RH (2014). The OPtimising HEalth LIterAcy (Ophelia) process: Study protocol for using health literacy profiling and community engagement to create and implement health reform. BMC Public Health.

[17] Beauchamp A, Batterham RW, Dodson S, Astbury B, Elsworth GR, McPhee C, Jacobson J, Buchbinder R, Osborne RH (2017). Systematic development and implementation of interventions to Optimise health literacy and access (Ophelia). BMC Public Health.

[18] Beauchamp A, Mohebbi M, Cooper A, Pridmore V, Livingston P, Scanlon M, Davis M, O’Hara J, Osborne R (2020). The impact of translated reminder letters and phone calls on mammography screening booking rates: Two randomised controlled trials. PLoS One.

[19] Bo A, Friis K, Osborne RH, Maindal HT (2014). National indicators of health literacy: Ability to understand health information and to engage actively with healthcare providers - a population-based survey among Danish adults. BMC Public Health.

[20] Boateng MA, Agyei-Baffour P, Angel S, Asare O, Prempeh B, Enemark U (n.d.)(pre-print under review) Co-Creation and Prototyping of An Intervention Focusing On Health Literacy In Management of Malaria At Community-Level In Ghana. Research Involvement and Engagement. doi:10.21203/rs.3.rs-123009/v1 10.1186/s40900-021-00302-0 PMC8340491 34353378

[21] Boateng MA, Angel S, Agyei-Baffour P, Enemark U (2020). Cultural adaptation and validation of the Ghanaian language (Akan; Asante Twi) version of the health literacy questionnaire. BMC health services research.

[22] Brazier JE, Harper R, Jones N, O'cathain A, Thomas K, Usherwood T, Westlake L (1992). Validating the SF-36 health survey questionnaire: New outcome measure for primary care. British medical journal.

[23] Buchbinder R, Batterham R, Elsworth G, Dionne CE, Irvin E, Osborne RH (2011). A validity-driven approach to the understanding of the personal and societal burden of low back pain: Development of a conceptual and measurement model. Arthritis research & therapy.

[24] Castillo-Díaz M, Padilla J-L (2013). How cognitive interviewing can provide validity evidence of the response processes to scale items. Soc Indic Res.

[25] Chapelle CA (2008). The TOEFL validity argument. Building a validity argument for the Test of English as a Foreign Language.

[26] Chapelle CA (2012). Validity argument for language assessment: The framework is simple … . Language Testing.

[27] Chapelle CA, Enright MK, Jamieson J (2010). Does an argument-based approach to validity make a difference?. Educational measurement: Issues and practice.

[28] Cook DA, Beckman TJ (2006). Current concepts in validity and reliability for psychometric instruments: Theory and application. The American journal of medicine.

[29] Cook DA, Brydges R, Ginsburg S, Hatala R (2015). A contemporary approach to validity arguments: A practical guide to Kane's framework. Med Educ.

[30] Cronbach L, Harris CW (1960). Validity. Encyclopedia of educational research: A project of the American Educational Research Association.

[31] Cronbach LJ, Thorndike RL, Angoff WH, Lindquist EF (1971). Test Validation. Educational Measurement. American Council on Education Washington.

[32] Cronbach LJ, Wainer H, Braun HI (1988). Five perspectives on validity argument. Test validity.

[33] Cronbach LJ, Meehl PE (1955). Construct validity in psychological tests. Psychological bulletin.

[34] Cureton E, Lindquist EF (1950). Validity. Educational measurement.

[35] Debussche, X., Caroupin-Soupoutevin, J., Balcou-Debussche, M., Fassier, M., Boegner, C., Hawkins, M., … Corbeau, C. (2021). Health literacy needs among migrant populations in France: validity testing and potential contribution of the Health Literacy questionnaire (HLQ). *Journal of Public Health (Berlin)*, 1–9. 10.1007/s10389-020-01423-8.

[36] Debussche X, Lenclume V, Balcou-Debussche M, Alakian D, Sokolowsky C, Ballet D, Elsworth GR, Osborne RH, Huiart L (2018). Characterisation of health literacy strengths and weaknesses among people at metabolic and cardiovascular risk: Validity testing of the health literacy questionnaire. SAGE Open Med.

[37] Edwards MC, Slagle A, Rubright JD, Wirth R (2018). Fit for purpose and modern validity theory in clinical outcomes assessment. Quality of life research.

[38] Elder C, Barber M, Staples M, Osborne RH, Clerehan R, Buchbinder R (2012). Assessing health literacy: A new domain for collaboration between language testers and health professionals. Language Assessment Quarterly.

[39] Elsworth GR, Beauchamp A, Osborne RH (2016). Measuring health literacy in community agencies: A Bayesian study of the factor structure and measurement invariance of the health literacy questionnaire (HLQ). BMC health services research.

[40] Gadermann AM, Guhn M, Zumbo BD (2011). Investigating the substantive aspect of construct validity for the satisfaction with life scale adapted for children: A focus on cognitive processes. Social Indicators Research.

[41] Gnanasakthy A, Barrett A, Evans E, D'Alessio D, Romano CD (2019). A review of patient-reported outcomes labeling for oncology drugs approved by the FDA and the EMA (2012-2016). Value Health.

[42] Goeman D, Conway S, Norman R, Morley J, Weerasuriya R, Osborne RH, Beauchamp A (2016). Optimising health literacy and access of service provision to community dwelling older people with diabetes receiving home nursing support. Journal of diabetes research.

[43] Goodwin BC, March S, Zajdlewicz L, Osborne RH, Dunn J, Chambers SK (2018). Health literacy and the health status of men with prostate cancer. Psycho-Oncology.

[44] Guillemin F, Bombardier C, Beaton D (1993). Cross-cultural adaptation of health-related quality of life measures: Literature review and proposed guidelines. Journal of clinical epidemiology.

[45] Guion RM (1977). Content validity—The source of my discontent. Appl Psychol Meas.

[46] Guion RM (1980). On Trinitarian doctrines of validity. Professional Psychology.

[47] Hatala R, Cook DA, Brydges R, Hawkins R (2015). Constructing a validity argument for the objective structured assessment of technical skills (OSATS): A systematic review of validity evidence. Advances in health sciences education.

[48] Hawkins M, Elsworth GR, Hoban E, Osborne RH (2020). Questionnaire validation practice within a theoretical framework: A systematic descriptive literature review of health literacy assessments. BMJ Open.

[49] Hawkins M, Elsworth GR, Osborne RH (2018). Application of validity theory and methodology to patient-reported outcome measures (PROMs): Building an argument for validity. Quality of life research.

[50] Hawkins M, Elsworth GR, Osborne RH (2019). Questionnaire validation practice: A protocol for a systematic descriptive literature review of health literacy assessments. BMJ Open.

[51] Hawkins M, Gill SD, Batterham R, Elsworth GR, Osborne RH (2017). The health literacy questionnaire (HLQ) at the patient-clinician interface: A qualitative study of what patients and clinicians mean by their HLQ scores. BMC health services research.

[52] Hawkins RE, Margolis MJ, Durning SJ, Norcini JJ (2010). Constructing a validity argument for the mini-clinical evaluation exercise: A review of the research. Academic medicine.

[53] Huang Y, Ruan T, Yi Q, Wang T, Guo Z (2019). The health literacy questionnaire among the aged in Changsha, China: Confirmatory factor analysis. BMC Public Health.

[54] Hubley AM, Zumbo BD (2011). Validity and the consequences of test interpretation and use. Social Indicators Research.

[55] Jessup RL, Osborne RH, Beauchamp A, Bourne A, Buchbinder R (2017). Health literacy of recently hospitalised patients: A cross-sectional survey using the health literacy questionnaire (HLQ). BMC health services research.

[56] Jessup RL, Osborne RH, Beauchamp A, Bourne A, Buchbinder R (2018). Differences in health literacy profiles of patients admitted to a public and a private hospital in Melbourne, Australia. BMC health services research.

[57] Jessup RL, Osborne RH, Buchbinder R, Beauchamp A (2018). Using co-design to develop interventions to address health literacy needs in a hospitalised population. BMC health services research.

[58] Kane M (2010). Validity and fairness. Lang Test.

[59] Kane M (2013). The argument-based approach to validation. School Psych Rev.

[60] Kane M, Mislevy R, Ercikan K, Pellegrino JW (2017). Validating score interpretations based on response processes. Validation of score meaning for the next generation of assessments.

[61] Kane MT (1990). An argument-based approach to validation. ACT Research Report Series. The American College Testing Program, Iowa.

[62] Kane MT (1992). An argument-based approach to validity. Psychological bulletin.

[63] Kane MT, Brennan RL (2006). Validation. Educational measurement, vol 4. ACE/Praeger series on higher education, vol 2, 4th edn. Rowman & Littlefield Publishers / Amer council Ac1 (pre Acq).

[64] Kane MT (2013). Validating the interpretations and uses of test scores. Journal of Educational Measurement.

[65] Kane MT (2016). Explicating validity. Assessment in Education: Principles, Policy and Practice.

[66] Kinsman L, Radford J, Elmer S, Ogden K, Randles S, Jacob A, Delphin D, Burr N, Goss M (2020). Engaging “hard-to-reach” men in health promotion using the OPHELIA principles: Participants' perspectives. Health promotion journal of Australia.

[67] Kolarčik P, Belak A, Osborne RH (2015). The Ophelia (OPtimise HEalth LIteracy and access) process. Using health literacy alongside grounded and participatory approaches to develop interventions in partnership with marginalised populations. European Health Psychologist.

[68] Kolarčik P, Cepova E, Geckova AM, Elsworth GR, Batterham RW, Osborne RH (2017). Structural properties and psychometric improvements of the health literacy questionnaire in a Slovak population. International journal of public health.

[69] Kroenke, K., Miksch, T. A., Spaulding, A. C., Mazza, G. L., DeStephano, C. C., Niazi, S. K., … Goyal, A. (2021). Choosing and using patient-reported outcome measures in clinical practice. *Archives of physical medicine and rehabilitation*. 10.1016/j.apmr.2020.12.033. 10.1016/j.apmr.2020.12.033 33713697

[70] Kwon, J. Y., Thorne, S., & Sawatzky, R. (2019). Interpretation and use of patient-reported outcome measures through a philosophical lens. *Qual Life Res, 28*, 629–639. 10.1007/s11136-018-2051-9. 10.1007/s11136-018-2051-9 30456714

[71] Landy FJ (1986). Stamp collecting versus science: Validation as hypothesis testing. The American psychologist.

[72] Lane S (2014). Validity evidence based on testing consequences. Psicothema.

[73] Leslie CJ, Hawkins M, Smith DL (2020). Using the health literacy questionnaire (HLQ) with providers in the early intervention setting: A qualitative validity testing study. International journal of environmental research and public health.

[74] Maindal HT, Kayser L, Norgaard O, Bo A, Elsworth GR, Osborne RH (2016) Cultural adaptation and validation of the health literacy questionnaire (HLQ): Robust nine-dimension Danish language confirmatory factor model. SpringerPlus 5 (1):1232. 10.1186/s40064-40016-42887-40069. 10.1186/s40064-016-2887-9 PMC4971008 27536516

[75] Marmot M, Bell R (2012). Fair society, healthy lives. Public Health.

[76] McClimans L (2010). A theoretical framework for patient-reported outcome measures. Theoretical medicine and bioethics.

[77] McCoach DB, Gable RK, Madura JP (2013). Instrument development in the affective domain.

[78] Messick S (1989). Meaning and values in test validation: The science and ethics of assessment. Educ Res.

[79] Messick S, Linn R (1989). Validity. Educational measurement.

[80] Messick S (1992). The interplay of evidence and consequences in the validation of performance assessments. ETS Research Report Series.

[81] Messick S (1993) Foundations of validity: Meaning and consequences in psychological assessment. ETS Res Rep Series 1993 (2):1-18

[82] Messick S (1998). Test validity: A matter of consequence. Soc Indic Res.

[83] Mislevy RJ (2003). Substance and structure in assessment arguments. Law Probabilty Risk.

[84] Mislevy RJ, Steinberg LS, Almond RGJMIr, perspectives (2003). On the structure of educational assessments. Measurement.

[85] Mitchell PM, Caskey FJ, Scott J, Sanghera S, Coast J (2020). Response process validity of three patient reported outcome measures for people requiring kidney care: A think-aloud study using the EQ-5D-5L, ICECAP-A and ICECAP-O. BMJ Open.

[86] Morris RL, Soh S-E, Hill KD, Buchbinder R, Lowthian JA, Redfern J, Etherton-Beer CD, Hill A-M, Osborne RH, Arendts G (2017). Measurement properties of the health literacy questionnaire (HLQ) among older adults who present to the emergency department after a fall: A Rasch analysis. BMC health services research.

[87] Moss PA, Girard BJ, Haniford LC (2006). Validity in educational assessment. Review of research in education.

[88] Mullan J, Burns P, Weston K, McLennan P, Rich W, Crowther S, Mansfield K, Dixon R, Moselen E, Osborne RH (2017). Health literacy amongst health professional university students: A study using the health literacy questionnaire. Education Sciences.

[89] Muscat DM, Song W, Cvejic E, Ting JHC, Medlin J, Nutbeam D (2020). The impact of the chronic disease self-management program on health literacy: A pre-post study using a multi-dimensional health literacy instrument. International journal of environmental research and public health.

[90] Nelson, E. C., Eftimovska, E., Lind, C., Hager, A., Wasson, J. H., & Lindblad, S. (2015). Patient reported outcome measures in practice. *BMJ*, *350*. 10.1136/bmj.g7818. 10.1136/bmj.g7818 25670183

[91] Nguyen TH, Park H, Han HR, Chan KS, Paasche-Orlow MK, Haun J, Kim MT (2015). State of the science of health literacy measures: Validity implications for minority populations. Patient Educ Couns.

[92] Nolte S, Osborne RH, Dwinger S, Elsworth GR, Conrad ML, Rose M, Härter M, Dirmaier J, Zill JM (2017). German translation, cultural adaptation, and validation of the health literacy questionnaire (HLQ). PLoS One.

[93] Onwuegbuzie AJ, Leech NL (2005). On becoming a pragmatic researcher: The importance of combining quantitative and qualitative research methodologies. International journal of social research methodology.

[94] Osborne RH, Batterham RW, Elsworth GR, Hawkins M, Buchbinder R (2013). The grounded psychometric development and initial validation of the health literacy questionnaire (HLQ). BMC Public Health.

[95] Padilla J-L, Benítez I (2014). Validity evidence based on response processes. Psicothema.

[96] Rademakers J, Waverijn G, Rijken M, Osborne R, Heijmans M (2020). Towards a comprehensive, person-centred assessment of health literacy: Translation, cultural adaptation and psychometric test of the Dutch health literacy questionnaire. BMC Public Health.

[97] Rheault H, Coyer F, Jones L, Bonner A (2019). Health literacy in indigenous people with chronic disease living in remote Australia. BMC health services research.

[98] Saleem, A., Steadman, K. J., Osborne, R. H., & La Caze, A. (2020). Translating and validating the health literacy questionnaire into Urdu: A robust nine-dimension confirmatory factor model. *Health promotion international*. 10.1093/heapro/daaa149. 10.1093/heapro/daaa149 33370429

[99] Sawatzky R, Chan EK, Zumbo BD, Ahmed S, Bartlett SJ, Bingham CO, Gardner W, Jutai J, Kuspinar A, Sajobi T (2017). Montreal accord on patient-reported outcomes (PROs) use series–paper 7: Modern perspectives of measurement validation emphasize justification of inferences based on patient reported outcome scores. Journal of clinical epidemiology.

[100] Shepard, L. A. (1993). Evaluating test validity. In: Darling-Hammond L (ed) review of research in education, vol 19. *Am Educ Res Asoc*, 405–450.

[101] Shepard LA (2016). Evaluating test validity: Reprise and progress. Assessment in Education: Principles, Policy & Practice.

[102] Sireci S, Faulkner-Bond M (2014). Validity evidence based on test content. Psicothema.

[103] Storey A, Hanna L, Missen K, Hakman N, Osborne RH, Beauchamp A (2020). The association between health literacy and self-rated health amongst Australian university students. Journal of health communication.

[104] Toulmin S (1988). The recovery of practical philosophy. The American Scholar.

[105] Toulmin SE (2003). The uses of argument.

[106] U.S. Food and Drug Administration, Center for Drug Evaluation and Research, Center for Biologics Evaluation and Research, Center for Devices and Radiological Health (2009). Guidance for industry: patient-reported outcome measures: use in medical product development to support labeling claims. Federal Register, vol 74. U.S.

[107] Urstad KH, Andenaes R, Wahl AK, Kvarme LG, Helseth S, Moum T (2020). The health literacy questionnaire: Initial validity testing in a Norwegian sample. HLRP.

[108] Wahl, A. K., Hermansen, Å., Osborne, R. H., & Larsen, M. H. (2020). A validation study of the Norwegian version of the health literacy questionnaire: A robust nine-dimension factor model. *Scandinavian Journal of Public Health, 49*(4), 471–478. 10.1177/1403494820926428. 10.1177/1403494820926428 PMC8135233 32508258

[109] Ware JE (2000). SF-36 health survey update. Spine.

[110] Ware Jr., J. E., Kosinski, M., & Keller, S. D. (1996). A 12-Item Short-Form Health Survey: construction of scales and preliminary tests of reliability and validity. *Med Care, 34*(3), 220–233. 10.1097/00005650-199603000-00003 8628042

[111] Zumbo BD, Chan EK (2014). Validity and validation in social, behavioral, and health sciences. Social indicators research series, vol 54.

[112] Zumbo BD, Hubley AM (2017). Understanding and investigating response processes in validation research, vol 69. Social Indicators Research Series.

